# Sulfonated Graphene Oxide Doped Imidazolium-Functionalized PVDF Ion Exchange Membrane with Enhanced Ion Conductivity

**DOI:** 10.3390/membranes16020055

**Published:** 2026-01-31

**Authors:** Jiangtao Yu, Wenkang Li, Wei Niu, Manman Zhang, Junqing Bai, Pengtao Li, Liang Wang, Yuqing Cui, Shuanfang Cui, Xueyan Que, Jun Ma, Long Zhao

**Affiliations:** 1 Yangling Hesheng Irradiation Technologies Co., Ltd., Xianyang 712000, China; yujiangtao@ssnhsit.com (J.Y.); niuwei@ssnhsit.com (W.N.); baijunqing@ssnhsit.com (J.B.); lipengtao@ssnhsit.com (P.L.); wangliang@ssnhsit.com (L.W.); cuiyuqing@ssnhsit.com (Y.C.); cuishuanfang@ssnhsit.com (S.C.); 2 School of Nuclear Science and Technology, University of Science and Technology of China, Hefei 230026, China; 3 State Key Laboratory of Advanced Electromagnetic Technology, School of Electrical and Electronic Engineering, Huazhong University of Science and Technology, Wuhan 430074, China; liwenkang@hust.edu.cn (W.L.); zhangmm@hust.edu.cn (M.Z.)

**Keywords:** sulfonated graphene oxide, proton conductivity, radiation grafting, ionic liquids, vanadium redox flow battery

## Abstract

A novel membrane was synthesized in this work by grafting 1-vinyl-3-ethylimidazolium tetrafluoroborate ([C_2_VIm][BF_4_]) onto a polyvinylidene fluoride (PVDF) backbone, followed by the introduction of a sulfonated graphene oxide (SGO) dispersion into the polymer solution. This composite was transformed into a composite proton-conducting membrane via a solution casting process and subsequently underwent protonation. Successful grafting was confirmed using analytical techniques including Fourier Transform Infrared Spectroscopy (FTIR), ^1^H Nuclear Magnetic Resonance (NMR) and X-ray Photoelectron Spectroscopy (XPS). Scanning Electron Microscopy with Energy Dispersive X-ray Spectroscopy (SEM-EDS) analysis verified the homogeneous distribution of the SGO filler. Analysis reveals that incorporating SGO as a filler substantially augments the performance of anion exchange membranes. Key enhancements include a tensile strength increase to 37.97 MPa, water uptake of 10.34%, an ion exchange capacity of 1.68 mmol/g, and the through-plane proton conductivity of 15.47 mS/cm. While vanadium permeability rose marginally to 2.02 × 10^−7^ cm^2^/min, it remains drastically lower than that of Nafion 115. The composite proton-conducting membrane also displayed robust chemical stability. The membrane was finally integrated into a vanadium redox flow battery (VRFB) for performance evaluation. At a current density of 100 mA/cm^2^, it exhibits a satisfactory coulombic efficiency (CE) of 97.84%, excellent capacity retention, and superior cycling stability. These results demonstrate that the PVDF-g-IL/SGO-based composite proton-conducting membrane is an ideal candidate material for vanadium flow battery applications.

## 1. Introduction

Among the various strategies to alleviate global energy shortages and environmental degradation, sustainable energy technologies demonstrate tremendous potential. Vanadium redox flow batteries (VRFBs) have become a prominent focus within energy storage research, particularly for grid-scale applications, owing to their substantial capacity, fast charging and discharging capabilities, outstanding efficiency, and enhanced safety profiles [1,2,3]. A significant advantage of these systems is their ability to facilitate the integration of storage on a large scale, making them suitable for uses such as stabilizing grid voltage, regulating frequency, and storing intermittent energy from renewable sources like solar and wind. Functioning as a critical element in the VRFB, the ion exchange membrane (IEM) serves the dual purpose of blocking the crossover of vanadium ions between electrochemical half-cells and enabling the ion conduction necessary to complete the circuit [4,5]. For optimal battery performance, an ideal membrane must exhibit high proton conductivity, minimal vanadium permeation, robust chemical and mechanical durability, and cost-effectiveness.

Many commercial membrane materials used in vanadium flow batteries exhibit insufficient vanadium permeation resistance and require further improvement in mechanical strength [6,7]. The Nafion series represents the most prevalent commercial choice, prized for its exceptional proton conductivity and strong mechanical characteristics [8,9]. Nevertheless, its prohibitive expense continues to hinder widespread practical adoption. Another drawback is the significant vanadium ion crossover in Nafion membranes after assembly, a phenomenon that induces self-discharge and reduces overall energy efficiency (EE) [10,11]. Consequently, a critical demand exists for the creation of new IEMs that integrate superior chemical resilience, high operational performance, and affordability for use in vanadium flow batteries. To address the shortcomings of Nafion, various alternative IEMs have been engineered. Broadly, these alternatives fall into two categories: non-fluorinated proton exchange membranes (PEMs) and anion exchange membranes (AEMs). Non-fluorinated PEMs, such as sulfonated poly(ether ether ketone) (SPEEK), offer a cost-effective alternative to Nafion with tunable properties and reasonably good proton conductivity [12]. However, their typically high water uptake and swelling can lead to elevated vanadium permeability, presenting a trade-off between conductivity and selectivity. To overcome this limitation, researchers have explored various modification strategies, such as introducing inorganic nanofillers (e.g., zirconium dioxide) to construct organic-inorganic hybrid membrane structures to simultaneously regulate the membrane’s hydrophilicity, dimensional stability, and ion selectivity [13]. On the other hand, AEMs often achieve remarkably low vanadium ion permeability. This is because their positively charged functional groups electrostatically repel the cationic vanadium species. The synthesis of most AEMs involves the attachment of these cationic groups onto a polymer backbone. From this group, membranes incorporating imidazolium functional groups demonstrate particularly promising results, a trait attributed to their distinctive π-conjugated five-membered ring architecture and inherent positive charge. This structure imparts a dual functionality: it facilitates the conduction of protons while simultaneously suppressing vanadium ion migration through the Donnan mechanism [14,15,16].

Radiation-induced grafting is extensively utilized as a viable method for fabricating IEMs used in electrochemical devices [17,18,19]. This approach presents considerable benefits compared to conventional techniques, distinguished by its straightforward and environmentally benign nature. It operates without catalysts or additives and initiates grafting reactions under ambient conditions. By modulating the radiation dosage and monomer concentration, the membrane’s composition and characteristics can be precisely tailored. The scalability of this technology for industrial manufacturing has also been well-demonstrated. Numerous studies confirm the effective synthesis of these membranes employing both hydrocarbon and fluorocarbon polymer backbones. Among these, fluorocarbon-based substrates are often favored owing to their enhanced thermal and chemical resilience, which is crucial for developing durable IEMs. Common base polymers include polyvinylidene fluoride (PVDF) [20,21], polytetrafluoroethylene (PTFE) [22], ethylene-vinyl alcohol copolymer (EVOH) [23] and ethylene-tetrafluoroethylene copolymer (ETFE) [24,25], grafted with assorted monomers. The resulting membranes demonstrate adequate chemical stability within systems like fuel cells and vanadium redox flow batteries. Nonetheless, their deployment in vanadium redox flow batteries is still limited by inherent drawbacks. For instance, while cation exchange membranes (CEMs) typically possess high conductivity, they also allow for significant vanadium ion crossover. Conversely, AEMs can inhibit vanadium ion diffusion effectively but are hampered by insufficient conductivity. Consequently, the creation of advanced IEMs that concurrently offer high proton conductivity and exceptional vanadium barrier properties is essential for the commercialization of vanadium flow batteries [26,27].

Studies demonstrate that the low conductivity of AEMs can be mitigated through the addition of diverse nanoscale fillers. These include SGO [28], graphene oxide [29], carbon nitride in graphite form [30], nanofibers of graphene [31], and sulfonated titanium dioxide [32]. SGO establishes continuous proton-conducting highways via the Grotthuss mechanism. This strategic design directly overcomes the core limitation of low proton conductivity in traditional AEMs within acidic VRFB electrolytes, effectively decoupling the ion transport mechanism. Moreover, SGO nanosheets not only serve as proton conductors but also function as structural reinforcements and hydrophilic nanospacers. They enhance the mechanical strength of the PVDF-based membrane and, more importantly, prevent the collapse of hydrophilic ion channels during drying. This significantly improves the accessibility of imidazole functional groups and the overall efficiency of ion transport [33].

In the present work, a critical consideration for VRFB membranes is their performance in the acidic electrolyte (3 M H_2_SO_4_), where protons constitute the majority of charge carriers. Consequently, the proton transport resistance of an AEM directly influences the ohmic losses and overall voltage efficiency of a VRFB system. This is a critical performance factor, despite the membrane’s principal role being the exchange of SO_4_^2−^/HSO_4_^−^ anions. Guided by the need to mitigate the inherent proton conductivity limitation of AEMs in acidic media, our material design incorporates a dual-strategy to enhance proton transport while preserving excellent anion-exchange capacity and vanadium rejection. Firstly, the imidazolium cation was strategically chosen as the functional group not only for its anion-exchange capability but also for its intrinsic potential to facilitate proton conduction. The imidazolium-functionalized composite membrane, after acid doping, operates effectively as a proton-exchange membrane in the acidic VRFB environment. Secondly, SGO was integrated into the matrix as a functional nanofiller to create dedicated, efficient pathways for proton hopping via the Grotthuss mechanism. Specifically, a pre-irradiation grafting method was initially utilized to attach the imidazolium ionic liquid (IL, [C_2_VIm][BF_4_]) onto a powder substrate of polyvinylidene fluoride (PVDF). Following grafting, the modified powder was dissolved and blended into a dispersion of SGO. The resulting mixture was solution-cast into a membrane and subsequently subjected to a final protonation treatment to obtain the composite proton-conducting membrane. The grafted samples were analyzed via several techniques: Fourier Transform Infrared Spectroscopy (FTIR), X-ray Photoelectron Spectroscopy (XPS), ^1^H Nuclear Magnetic Resonance (^1^H NMR), Thermogravimetric Analysis (TGA), and Scanning Electron Microscopy-Energy Dispersive Spectroscopy (SEM-EDS). A critical assessment was conducted on the membrane’s ion exchange capacity, rate of water uptake, proton conductivity and VRFB system performance. The structure of this composite membrane successfully overcomes the performance drawbacks of AEM’s poor proton conductivity and CEM’s high vanadium permeability, offering a promising, innovative solution for next-generation vanadium battery membranes.

## 2. Experimental

### 2.1. Materials

Of the materials utilized, polyvinylidene fluoride powder (Solef 6020) was sourced from SOLVAY lnc. The compound [C_2_VIm][BF_4_] was supplied by the Lanzhou Institute of Chemical Physics. N-Methyl-pyrrolidone (NMP) and other general chemical reagents were procured from Sinopharm Chemical Reagent Co., Ltd. SGO (average particle size: approximately 26.7 nm) was obtained from Shenzhen Angxing Technology Co., Ltd., while vanadium (V) sulfate (VOSO_4_) was provided by Wuxi Zhanwang Chemical Reagent Co., Ltd. A Nafion 115 membrane was supplied by DuPont. Every chemical was employed in its received state, with no additional purification steps undertaken.

### 2.2. Radiation-Induced Graft Copolymerization of [C_2_VIm][BF_4_] into PVDF

The PVDF-g-IL powder with a grafting yield of 20% was prepared by the pre-irradiation grafting technique. Firstly, pure PVDF powder was placed in polyethylene bags and hermetically sealed under vacuum. This sealed material was then subjected to electron beam irradiation, delivered at a total dosage of 100 kGy (20 kGy per pass, with five passes in total), utilizing an accelerator from Wasik Associates INC. Concurrently, a 25 wt% aqueous solution of the monomer [C_2_VIm][BF_4_] was prepared and purged with nitrogen gas for a duration of half an hour. The irradiated powder was subsequently combined with this monomer solution, and the reaction was allowed to proceed at 60 °C over a 24 h period. After the grafting was complete, the resultant PVDF-g-IL powder underwent a purification process to eliminate impurities, which involved successive washes with deionized water and ethanol. The final product was then dried at 60 °C until a constant mass was achieved.

### 2.3. Fabrication of the Membranes

**Scheme 1:**
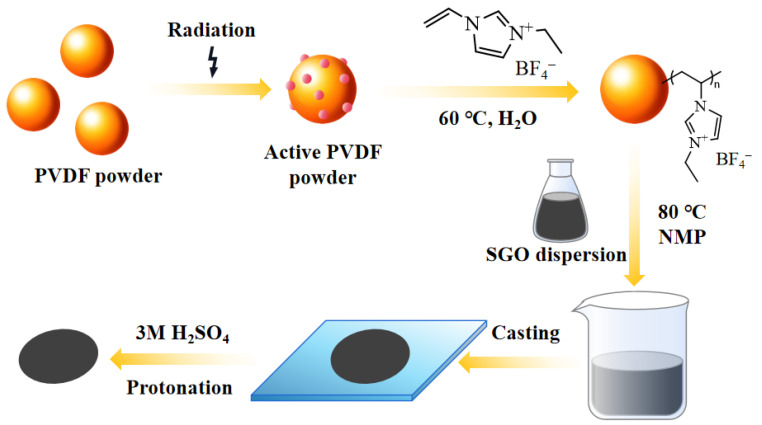
The fabrication steps of the grafted powders and the membrane.

To prepare the casting solution, PVDF-g-IL powder was first dissolved in NMP at 80 °C. This solution was subsequently blended with a separate NMP dispersion containing SGO. The overall solid concentration of the grafted polymer powder was maintained at 10 wt%. The incorporated SGO constituted 0.5, 1, 2.5, or 5 wt% relative to the mass of the PVDF-g-IL powder. The resulting mixture was then cast onto an even, clean glass substrate. The substrate was transferred to a blast drying oven and dried at 60 °C for 12 h to ensure full solvent evaporation. After drying, the adhered film was delaminated by submerging the glass plate in water. The peeled film was subsequently protonated by immersion in a 3 M sulfuric acid (H_2_SO_4_) solution for 72 h. Finally, the protonated films were kept hydrated in water until further use. For comparative analysis, a pure PVDF-g-IL film was also fabricated following an identical procedure. An overview of the entire preparation process for the grafted powders and the composite proton-conducting membrane is provided in Scheme 1.

### 2.4. Characterization

Fourier-transform infrared (FT-IR) spectroscopy was carried out on a Bruker VERTEX 70 spectrometer (Germany), scanning a spectral region from 400 to 4000 cm^−1^ with a resolution set to 2 cm^−1^. For nuclear magnetic resonance (NMR) analysis, the PVDF-g-IL powder was dissolved in deuterated dimethyl sulfoxide (DMSO-d6) to prepare the sample solution. NMR spectra were subsequently acquired on an AscendTM 600 MHz spectrometer (Bruker, Germany) operating in transmission mode. X-ray photoelectron spectroscopy (XPS) was performed with a Kratos Analytical AXIS-UItra instrument. To examine membrane surface morphology, field emission scanning electron microscopy coupled with energy dispersive X-ray spectroscopy (SEM-EDS) was employed using a Hitachi SU8010 system (Japan). The mechanical properties of both the pristine PVDF membrane and the fabricated composite proton-conducting membranes were determined with a SEMtester 100 (Milliren Technologies Inc., Newburyport, MA, USA), applying a 100 N load. All membranes were cut into standardized strips measuring 5 cm by 1 cm prior to testing.

### 2.5. Antioxidant Testing

The electrolyte in VRFBs contains highly oxidative pentavalent vanadium ions, creating a strongly oxidizing environment for the separator. Consequently, the chemical stability of the separator significantly impacts its performance and operational lifespan. To assess antioxidant properties, pre-treated membrane specimens, each measuring 5 cm × 5 cm, were first sectioned and placed in a drying oven maintained at 80 °C. Drying was continued until the mass, measured with an analytical balance, stabilized, defined as two consecutive measurements remaining essentially unchanged. These dried samples were then fully immersed in a 1.5 M VO_2_^+^ solution prepared in aqueous 3 M sulfuric acid. Following a seven-day immersion period at ambient temperature, the membranes were retrieved. They were rinsed thoroughly with deionized water until the effluent attained a neutral pH, verified as 7 using universal pH paper. The cleaned membranes underwent a second drying cycle in the oven under identical temperature conditions, with the same mass stability criterion applied to confirm complete dryness before their final mass was recorded. To evaluate the degree of oxidation, the concentration of VO_2_^+^ ions in the solution was analyzed with a UV spectrophotometer and compared against a control solution that contained no membrane.

### 2.6. Ion Exchange Capacity, Water Uptake, Swelling Ratio and Proton Conductivity of the PVDF-g-IL/SGO IEM

The ion exchange capacity (IEC) was determined through an acid–base titration technique. First, dried membrane samples were allowed to equilibrate in a 0.5 M sodium hydroxide (NaOH) solution for a period of 24 h. Subsequently, the resulting solution was titrated in reverse using a 0.5 M hydrochloric acid (HCl) solution to reach a neutral pH endpoint. The IEC value was then calculated based on the titration data, utilizing Equation (1).

(1) $$\mathrm{IEC}=\frac{C(\mathrm{NaOH})\times V(\mathrm{NaOH})-C(\mathrm{HCl})\times V(\mathrm{HCl})}{W_{\mathrm{dry}}}$$

where C(HCl) and C(NaOH) are the concentrations (mol L^−1^) of the initial HCl and NaOH solutions, respectively; V(HCl) and V(NaOH) are the volumes of the used HCl solution by back titration and the initial NaOH solution, respectively; W_dry_ is the weight (g) of the dry membrane.

To determine the water uptake (WU), a gravimetric analysis was performed. The dry mass of each membrane was first recorded. Subsequently, the samples were submerged in deionized water and allowed to soak at ambient temperature for 24 h. Following this hydration period, the membranes were removed from the aqueous medium. Any residual surface moisture was carefully blotted away, and the hydrous membranes were immediately reweighed to obtain their wet mass. For evaluating the acid uptake (AU), the membranes were soaked in 3 M H_2_SO_4_ for 24 h. Then, the membranes were dried in the vacuum oven at 80 °C for 24 h to remove the water and measured their mass (W_acid_). The WU and AU could be calculated as Equations (2) and (3), respectively.

(2) $$\mathrm{WU}=\frac{W_{\mathrm{wet}}-W_{\mathrm{dry}}}{W_{\mathrm{dry}}}\times 100\%$$

(3) $$\mathrm{AU}=\frac{W_{\mathrm{acid}}-W_{\mathrm{dry}}}{W_{\mathrm{dry}}}\times 100\%$$

where W_dry_ and W_wet_ are the weights (g) of the membranes before and after soaking.

The swelling ratio (SR) was ascertained through a volumetric methodology. The initial dimensions—specifically the length, width, and thickness—of every dry membrane sample were measured. These specimens were then fully submerged in deionized water and left to soak for a period of 24 h under ambient conditions. Post-hydration, the membranes were extracted from the water. After meticulously blotting any superficial liquid, their swollen dimensions were promptly measured again. The SR was subsequently computed from the observed change in volume by applying Equation (4).

(4) $$\mathrm{SR}=\frac{l_{2}\times d_{2}\times h_{2}-l_{1}\times d_{1}\times h_{1}}{l_{1}\times d_{1}\times h_{1}}\times 100\%$$

where l_1_, d_1_, and h_1_ represent the length, width, and height of the membrane before immersion treatment, and l_2_, d_2_, and h_2_ represent the corresponding dimensions after immersion treatment.

The through-plane proton conductivity of the membranes was measured by electrochemical impedance spectroscopy (EIS) using a CHI660E electrochemical workstation. In accordance with national standard NB/T42080-2023, prior to measurement, the membrane sample shall be fully immersed in a 3 M H_2_SO_4_ solution for 24 h. The test apparatus is illustrated in Figure S1. The effective electrode area (S) is determined by the mold geometry used in the symmetrical double-electrode cell. The effective area is 1 cm^2^ (1 cm × 1 cm). The membrane thickness (L) shall be determined by taking measurements at no fewer than three arbitrary positions on the sample using a digital micrometer, after which the representative mean thickness is calculated. During testing, the entire apparatus is placed in a thermostatic chamber maintained at a constant temperature of 25 ± 0.5 °C. An alternating current disturbance of 5 mV is applied within a frequency range of 1 to 10^5^ Hz. Both sides of the cell are filled with 3.0 M SA solution. The proton conductivity (σ, mS cm^−1^) is calculated from the obtained impedance data using Equation (5):

(5) $$\sigma =\frac{L}{(R_{2}-R_{1})S}$$

where L is the thickness (cm) of the membrane, S is a fixed value representing the effective area of the membrane, and the resistance values measured before and after installing the membrane are labeled as R_1_ and R_2_, respectively.

### 2.7. Vanadium Permeability and Selectivity

Within VRFBs, the extent of vanadium ion permeation is a critical performance indicator. A lower permeation rate directly mitigates self-discharge, which in turn supports the preservation of the system’s total capacity. The vanadium ion permeability (P) of the prepared composite proton-conducting membranes and a Nafion 115 reference was assessed using a diffusion cell fabricated from PTFE. This apparatus consisted of two 50 mL half-cells, each featuring a 1.5 cm diameter circular opening. Prior to the evaluation, the composite proton-conducting membranes were protonated by soaking them in a 3 M H_2_SO_4_ solution for 6 h. A membrane was then securely clamped between the two halves of the cell. One reservoir was charged with 50 mL of a solution containing 1.5 M VOSO_4_ in 3 M H_2_SO_4_, and the opposing reservoir was filled with 50 mL of 1.5 M MgSO_4_ dissolved in an identical 3 M H_2_SO_4_ medium. At predetermined time intervals, aliquots were extracted from the reservoir containing MgSO_4_ to monitor the temporal change in vanadium concentration. The quantity of vanadium ions present in these samples was quantified using an Agilent 5110 ICP-OES (Agilent, Santa Clara, CA, USA) instrument (inductively coupled plasma optical emission spectrometer). The permeability coefficient and selectivity were ultimately determined by substituting the data into Equations (6) and (7) [34].

(6) $$P=\frac{\mathrm{LV}}{A(C_{a}-C_{b}\left(t)\right)}(\frac{d(C_{b}\left(t)\right)}{\mathrm{dt}})$$

(7) $$S=\frac{\sigma }{P}$$

where L is the thickness (cm) of the membrane, V is the volume (mL) of the left cell, A is the effective area (cm^2^) of the membrane, C_a_ and C_b_ (t) are the concentrations (mol L^−1^) in the left cell and right cell at time t (min), respectively. P is the vanadium permeability of the membrane. σ represents conductivity, and S is selectivity.

### 2.8. The Performance of VRFBs Assembled with the Membranes

The battery assembly was constructed by layering each membrane with two carbon felt electrodes, two graphite plates, and two copper plates. This stack was then secured between two external casings. Within the resulting cell, the membrane’s active surface area measured 4 cm^2^. Both the positive and negative half-cells were filled with 1.7 M V^3+^/V^4+^ in 3.0 M H_2_SO_4_. The vanadium electrolyte was prepared by electrolytic dissolution of VOSO_4_ in sulfuric acid. The voltage cut-off limits were set at 0.8 V and 1.65 V. 20 mL for each half-cell. The electrolyte was circulated using peristaltic pumps. All cell tests were conducted at a controlled room temperature of 25 ± 2 °C. Two peristaltic pumps circulated the electrolytes at a flow rate of 50 mL min^−1^. To evaluate the open-circuit voltage (OCV), vanadium flow batteries assembled with either the anion exchange membranes or a Nafion 115 benchmark were initially charged at a constant current density of 100 mA cm^−2^ until fully charged, after which they were allowed to discharge passively. Charge–discharge cycling performance was assessed by operating the batteries across a range of current densities from 40 to 120 mA cm^−2^. A multi-channel battery analyzer (BTS-8, Shenzhen Kejing Star Technology Co., Ltd., Shenzhen, China) was employed to collect all performance data. The voltage thresholds for terminating the charge and discharge cycles were established at 1.65 V and 0.8 V, respectively. Finally, the CE, EE, and voltage efficiency (VE) were determined using Equations (8)–(10).

(8) $$\mathrm{CE}=\frac{{\mathrm{total}\;\mathrm{charge}}_{\mathrm{discharge}}}{{\mathrm{total}\;\mathrm{charge}}_{\mathrm{charge}}}\times 100\%$$

(9) $$\mathrm{EE}=\frac{{\mathrm{energy}\;\mathrm{density}}_{\mathrm{discharge}}}{{\mathrm{energy}\;\mathrm{density}}_{\mathrm{charge}}}\times 100\%$$

(10) $$\mathrm{VE}=\frac{\mathrm{EE}}{\mathrm{CE}}\times 100\%$$

## 3. Results and Discussion

### 3.1. Characterization of the Prepared PVDF-g-IL Powders and PVDF-g-IL/SGO Composite Proton-Conducting Membranes

**Figure 1:**
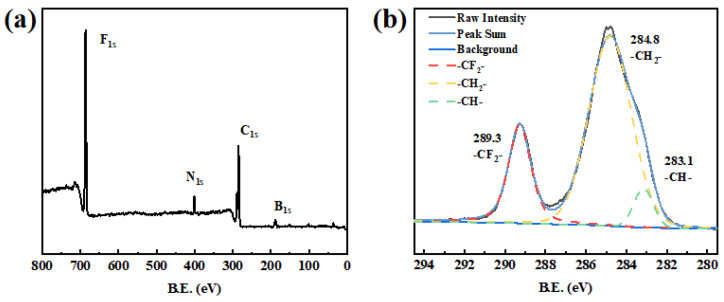
(**a**) XPS wide scan analysis and (**b**) the C_1s_ core-level spectra of PVDF-g-IL membrane.

Employing a straightforward pre-irradiation technique, imidazolium-based ILs were immobilized onto PVDF powder via covalent grafting. Earlier research has detailed how varying the extent of IL grafting influences the properties of membranes. For this investigation, all further tests were performed using the established optimum grafting degree of 20% [35]. This specific degree provides a high density of anion-exchange groups while ensuring the complete solubility of the polymer necessary for fabricating homogeneous membranes. Infrared spectral data (Figure S2) exhibited skeletal stretching vibrations of the imidazolium ring at 1573 cm^−1^ and 1552 cm^−1^ following grafting. A distinctive B–F absorption peak was also observed at 1084 cm^−1^. The success of the grafting process was further verified by ^1^H NMR spectroscopy (Figure S3), which displayed signals at 7.54 ppm, 7.79 ppm, and 9.06 ppm. These shifts are attributable to hydrogens within distinct chemical settings on the imidazolium cation. XPS is a standard analytical method for characterizing polymer surfaces. The XPS survey spectrum acquired for the PVDF-g-IL film is presented in Figure 1. The successful grafting of the IL monomer onto the membrane is confirmed by the emergence of distinct N_1s_ and B_1s_ peaks, as visible in Figure 1a. Furthermore, the high-resolution C_1s_ core level spectrum of this same film is displayed in Figure 1b. Deconvolution of this signal reveals three constituent peaks at binding energies of 283.1 eV, 284.8 eV, and 289.3 eV. These components are assigned to carbon atoms in the -CH- groups of the imidazole aromatic rings, the -CH_2_-, and the -CF_2_- units, respectively [36,37]. Together, these analytical results confirm the effective covalent attachment of the IL species.

**Figure 2:**
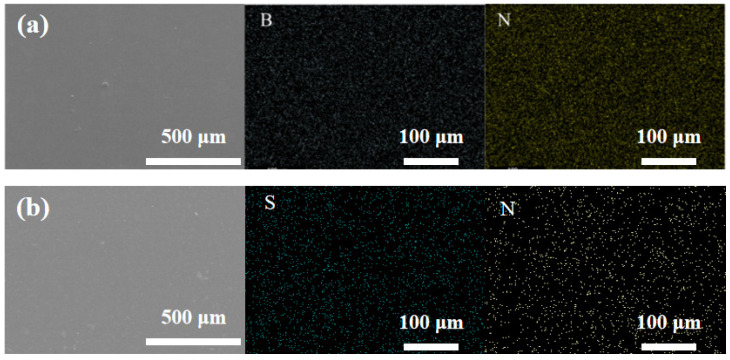
The SEM-EDS images of the (**a**) PVDF-g-IL and (**b**) PVDF-g-IL/5%SGO membranes.

The synthesized PVDF-g-IL powder was first dissolved in NMP at 80 °C, then cooled to ambient temperature. In a separate step, SGO was dispersed into NMP via probe ultrasonication at 0 °C. These two prepared mixtures were then combined and agitated vigorously to produce a homogeneous casting solution. The mass fraction of SGO in these solutions was set at 0.5, 1, 2.5, and 5 wt% relative to the grafted polymer powder. The final casting solution’s concentration was carefully adjusted to achieve a consistent film thickness of approximately 120 μm. As illustrated in Figure 2, the SEM-EDS micrographs depict the PVDF-g-IL membrane and the PVDF-g-IL/SGO composite proton-conducting membrane. Figure 2a reveals a homogeneous spatial distribution of boron (B) and nitrogen (N) elements throughout the membrane. This uniformity suggests that the pre-irradiation technique facilitates consistent graft copolymerization of ILs onto the PVDF matrix. Such even modification is critical, as it prevents the formation of irregular structures that could disrupt ion-conducting channels, elevate electrical resistance, and consequently diminish overall proton conductivity. Following the incorporation of SGO, sulfur (S) signals showed a homogeneous distribution throughout the PVDF-g-IL/5%SGO membrane, as observed in both surface (Figure 2b) and cross-sectional (Figure S4) views. This homogeneous distribution indicates that the SGO is effectively prevented from aggregating through π-π stacking interactions. Thereby, it avoids the creation of obstructive agglomerates that could act as barriers to ion transport or establish non-conductive regions within the membrane.

The fixed imidazolium cations create anion-exchange sites. In the acidic vanadium electrolyte, the dominant charge carriers for this pathway are HSO_4_^−^ and SO_4_^2−^ ions. These anions migrate through the hydrophilic domains of the membrane primarily via the Vehicle mechanism, where they diffuse as hydrated species. This pathway is primarily responsible for maintaining charge balance and is crucial for the membrane’s ability to prevent cross-migration of vanadium cations. Additionally, the incorporated SGO introduces sulfonic acid groups (-SO_3_H) that form efficient proton-conducting pathways. Protons travel along these SGO networks via the Grotthuss (hopping) mechanism, which is inherently faster than the vehicle mechanism. This pathway is specifically designed to facilitate the transport of the majority cation in the acidic electrolyte (H^+^), thereby significantly reducing the membrane’s area-specific resistance.

### 3.2. The Performance of the Composite Proton-Conducting Membranes

#### 3.2.1. Mechanical Characteristics

**Figure 3:**
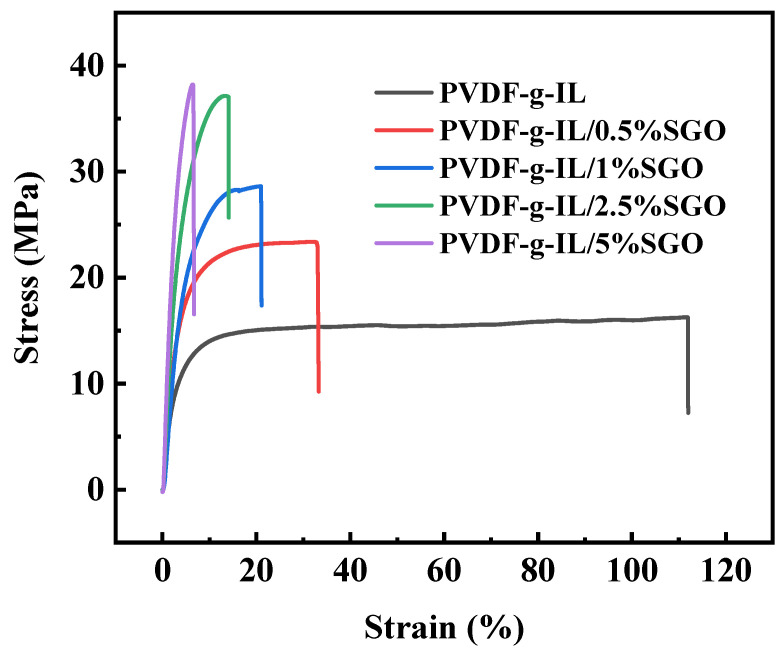
Mechanical properties of the PVDF-g-IL membrane and the PVDF-g-IL/SGO membranes.

The long-term functionality of VRFBs is heavily dependent on the mechanical properties of the membranes. For practical use, the membrane requires robust mechanical strength to endure the forces exerted by flowing electrolyte and must be durable enough to reduce the frequency and expense of replacements. To assess these properties under simulated operating conditions, the composite proton-conducting membrane samples were first soaked in deionized water for a full day before analysis. Their tensile strength and elongation at break were then measured, and the corresponding data are presented in Figure 3 and Figure S5. The results demonstrate a clear trend: as the concentration of SGO is raised, the composite proton-conducting membranes’ tensile strength is enhanced, but their elongation at break is reduced. This correlation is attributed to the incorporation of rigid, well-dispersed SGO nanosheets into the polymer network. These sheets act as a reinforcing filler, absorbing the applied stress and thereby substantially improving the composite’s resistance to deformation and fracture, which is reflected in the higher tensile strength. When the doping concentration of SGO reaches 0.5 wt%, its tensile strength already surpasses that of Nafion 115. Conversely, the presence of these fillers constrains the mobility of the polymer chains, reducing their flexibility and capacity to stretch. Thus, a much greater force is needed to cause the material to fail (indicating high strength), but when this ultimate stress is reached, the material breaks in a brittle manner with little drawn-out deformation (indicating low elongation).

#### 3.2.2. Antioxidant Properties

The operational longevity of VRFBs is compromised by the presence of strongly oxidizing vanadium ions, which aggressively assault the composite membranes. This chemical attack induces an oxidative breakdown of the material, leading to a decline in both its mechanical integrity and the overall efficacy of the battery. Consequently, developing composite membranes with outstanding resistance to oxidation is vital for ensuring the durable and stable performance of VRFB systems. Chemical stability was assessed using gravimetric analysis of Nafion 115, PVDF-g-IL, and PVDF-g-IL/SGO membranes. Each sample was submerged for 7 days in a 1.5 M VO_2_^+^ solution prepared in aqueous 3 M sulfuric acid, and the subsequent change in its mass was recorded (Table 1). The data reveal that Nafion 115 experienced a comparatively smaller reduction in weight, underscoring its greater chemical resilience relative to the grafted PVDF-g-IL variants. A notable observation is that, while the PVDF-g-IL series membranes demonstrate greater mass loss compared to Nafion 115, essential functional characteristics are retained. Key spectroscopic signatures, such as the C=N stretching vibration of the imidazolium group, show minimal change, and ionic conductivity remains above 90% of the initial value (Figure S6). This indicates that although some physical degradation occurs, the core ionic functionality and conductive networks within the material are largely preserved. These results underscore the inherent stability of the PVDF matrix and the robust contribution of the imidazolium functional groups.

#### 3.2.3. Water Uptake Rate, Acid Uptake Rate and Volume Swelling Rate

Key characteristics of the examined membranes, including water uptake, acid uptake, and volume swelling, are summarized in Table 2. A consistent thickness of roughly 120 μm was achieved for the PVDF, PVDF-g-IL, and PVDF-g-IL/SGO membranes by carefully regulating the polymer solution concentration during fabrication. The incorporation of SGO led to a consistent increase in acid uptake (in 3 M H_2_SO_4_), water uptake (in deionized water), and the associated swelling ratio for the composite membranes. This trend stems from the enhanced hydrophilicity resulting from the synergistic interaction between the sulfonic acid groups of SGO and the protonated imidazolium cations. Concurrently, the incorporation of SGO alters the membrane’s microstructure, thereby facilitating easier contact between the imidazole groups and the sulfuric acid solution. While water uptake indicates the intrinsic hydrophilic potential, the acid uptake directly reflects the membrane’s practical proton-exchange capacity in the operating environment, as the absorbed acid provides both the charge carriers (H^+^) and the essential hydrated pathways for conduction. However, increased adsorption can also induce structural changes within the membrane, which may affect its operational stability. Thus, evaluating the swelling behavior is essential. The findings reveal that the base PVDF-g-IL membrane displays swelling characteristics similar to those of Nafion 115. On the other hand, membranes modified with SGO show noticeably higher swelling rates, which can be primarily ascribed to the enhanced hydrophilicity imparted by the additive. Even with this increase, the PVDF-g-IL/5%SGO membrane registered a moderate swelling rate of 12.85%, which remains within an acceptable range for use in operational VRFB systems.

#### 3.2.4. Ion Exchange Capacity and Ion Conductivity

**Figure 4:**
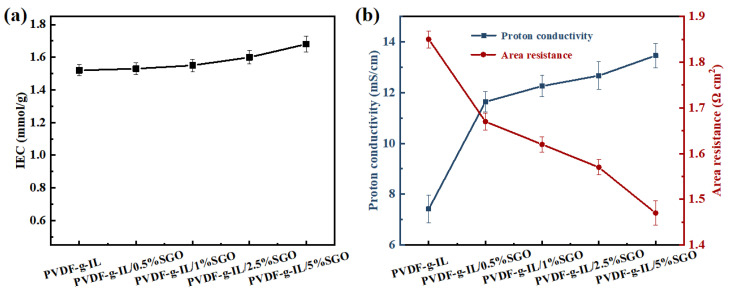
(**a**) The IEC of PVDF-g-IL and PVDF-g-IL/SGO composite proton-conducting membranes; (**b**) proton conductivity and area resistance of the PVDF-g-IL/SGO membranes.

The IEC for the PVDF-g-IL and PVDF-g-IL/SGO composite proton-conducting membranes is presented in Figure 4a. As illustrated, the IEC of the PVDF-g-IL composite proton-conducting membranes increases progressively with higher concentrations of SGO. This enhancement effect arises from the following mechanism. Upon introduction of SGO nanosheets, they function as rigid hydrophilic nanospacers, preserving the porosity and accessibility of the hydrophilic regions containing imidazolium cations. This facilitates more complete ion exchange during IEC titration, effectively exposing imidazolium sites that would otherwise remain inaccessible in the pure membrane. Moreover, SGO nanosheets create additional interfaces and potential continuous pathways within the membrane. This enhanced connectivity facilitates the penetration of titrant ions into the membrane, enabling more thorough ion exchange reactions and yielding higher measured IEC values. Therefore, a direct relationship exists between the SGO content and the measured IEC in the PVDF-g-IL/SGO membranes. Notably, the ion exchange capacity (IEC) of the PVDF-g-IL/SGO composite membrane surpassed that of the Nafion 115 reference membrane (0.93–0.95 mmol/g). It is important to distinguish that the ion exchange capacity serves as a measure of the total quantity of sites available for proton adsorption within the material. In contrast, proton conductivity is governed by the mobility of protons and their ability to traverse the membrane’s internal structure.

Consequently, the membrane’s impedance was characterized. The corresponding electrochemical impedance spectroscopy (EIS) data, fitted using an equivalent circuit model, are presented in Figure S7. The ionic conductivity calculated from these EIS measurements is displayed in Figure 4b. As depicted in Figure 4b, the ionic conductivity of the ionic-liquid-grafted PVDF-g-IL ion-exchange membrane attained 7.42 mS/cm. This phenomenon arises from the acidification process in 3 M H_2_SO_4_, which fully protonates the imidazole nitrogen atoms, converting the membrane into a polycationic network bearing fixed positive charges (imidazolium cations). Within this structure, protons serve as the primary mobile charge carriers, thereby imparting pronounced conductive properties to the PVDF-g-IL membrane. Moreover, proton conductivity exhibits a significant positive correlation with increasing SGO content, a trend that aligns with the concurrently measured rise in IEC. Correspondingly, the membrane’s sheet resistance decreases substantially with higher SGO doping concentrations. The performance enhancement observed in our PVDF-g-IL/SGO composites aligns with the widely reported trend for organic-inorganic hybrid membranes based on other polymer matrices (e.g., SPEEK) and diverse nanofillers. This consistency further confirms that synergistically optimizing multifunctional properties—such as enhanced mechanical properties and optimization of proton conduction pathways—through a rationally designed hybrid architecture is a broadly effective and promising strategy for developing advanced VRFB membranes [13].

This enhancement is rationalized by the formation of dynamic acid–base ion pairs between the basic imidazolium cations of the polymer matrix and the acidic sulfonic acid groups (-SO_3_H) on the SGO nanosheets. Crucially, within the context of an anion-exchange membrane, this interaction does not lead to a static neutralization [38,39]. Instead, it creates a unique interfacial nano-environment that facilitates ion conduction through two synergistic effects. First, the acid–base pairs between the basic imidazolium cations of the polymer matrix and the acidic -SO_3_H on the SGO nanosheets create a dynamic, proton-sharing interface that establishes a low-energy-barrier pathway for the Grotthuss (hopping) mechanism. This interface may stabilize transition states and promote a Grotthuss-type (hopping) mechanism for the ion. Second, the two-dimensional, highly hydrophilic SGO nanosheets and these interfacial ion-pair regions act as organizers for enhanced microphase separation. They promote the formation of interconnected, nano-sized hydrophilic domains within the polymer matrix. These domains, enriched with ionic species and water, function as efficient highways for ion and water transport, significantly reducing the diffusional resistance for migrating ions. With increasing SGO content, these individual, organized nanosheets come into closer proximity, eventually percolating to form a continuous network of well-hydrated, ion-conducting pathways throughout the membrane. The establishment of this percolated network is a key factor behind the substantial improvement in ion conductivity. It is important to note that the proposed mechanism—wherein SGO acts not as a direct charge carrier donor but as a nanostructurant and ionic environment modifier that enhances ion mobility via ion-pair interfaces and percolated pathways—is a rational interpretation based on the consistent trends in our conductivity, ion exchange capacity, and morphological data. It is supported by the analogous principle of ion-pair facilitated transport noted in the literature [35,36], although here it is applied to an anion-conducting system. This mechanism, while strongly suggested by our findings, warrants further validation through in situ spectroscopic or computational studies in future work.

As depicted in Figure 4, the material’s capacity for proton transport remains constrained by its intrinsic characteristics, notwithstanding its high IEC. The incorporation of rigid, hydrophilic SGO nanosheets disturbs the continuity of the hydrophobic PVDF matrix. This structural disruption mitigates the collapse of ionic domains and simultaneously establishes a more extensive network of continuous channels to facilitate the movement of both water molecules and protons. The measured increase in the composite membrane’s IEC provides direct confirmation of this effect. Consequently, a substantial enhancement in proton conductivity is maintained relative to the unmodified PVDF-g-IL membrane. However, the proton conductivity of PVDF-g-IL/SGO composite proton-conducting membranes is lower than that of Nafion 115 (43.55 mS/cm). As a CEM, it possesses abundant and continuous -SO_3_H and hydrophilic channels for water molecules. Within these channels, water molecules assemble into an extensive hydrogen-bonding network. This arrangement permits protons to traverse at exceptionally high rates, primarily through the Grotthuss mechanism. The operational conditions typical for AEMs are not conducive to the Grotthuss mechanism, which leads to an intrinsic constraint on their proton conduction capabilities. To counteract this limitation, the membrane is functionalized with polymerized imidazole chains, which are proficient in facilitating proton transport, and is further modified with SGO. A separate and considerable benefit of our fabricated composite proton-conducting membranes is their strong resistance to vanadium ion diffusion. Consequently, this property plays a crucial role in mitigating capacity fade within the battery system and leads to a substantial reduction in the expenses associated with electrolyte replenishment.

#### 3.2.5. Permeability of Vanadium Ions

**Figure 5:**
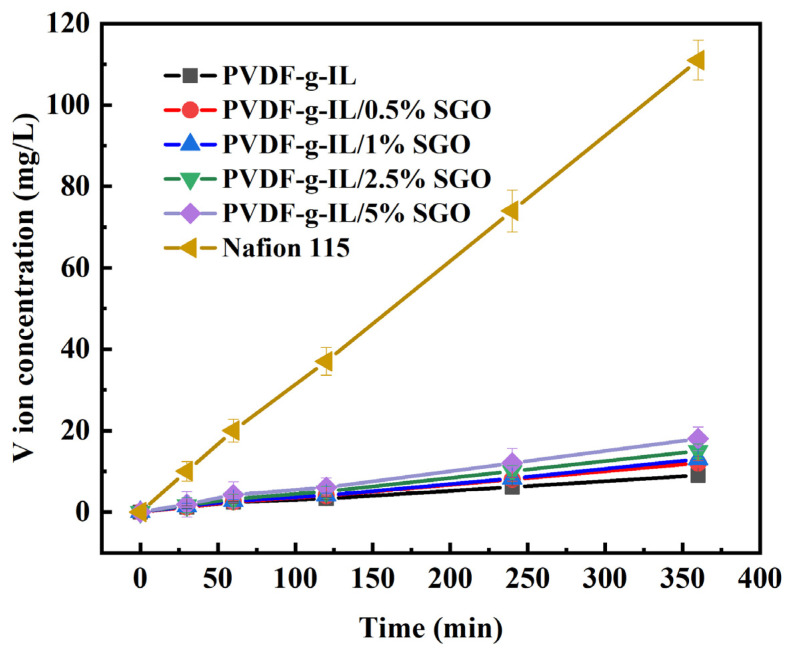
The vanadium ion permeability of the PVDF-g-IL and PVDF-g-IL/SGO membranes and Nafion115.

In vanadium flow battery systems, the permeability of vanadium ions stands as a paramount performance metric. Reducing this permeability minimizes self-discharge and thereby helps retain overall battery capacity. As shown in Figure 5, the measured VO^2+^ permeability across all evaluated composite proton-conducting membranes was significantly lower than that of the Nafion 115 benchmark. Specifically, the PVDF-g-IL membrane demonstrated a vanadium ion permeability of 0.95 × 10^−7^ cm^2^/min, a value that is an order of magnitude lower, approximately one-twelfth, than that of Nafion 115. However, composite proton-conducting membranes with higher SGO loadings exhibit reduced ability to block vanadium ions, as shown in Table 3. A plausible explanation for this trend lies in the associated increase in the membrane’s swelling rate (Table 2). The introduction of greater amounts of the hydrophilic nanofiller induces more expansive swelling, which can generate wider and more numerous aqueous pathways through the polymer matrix. These enlarged channels facilitate the crossover of vanadium ions, thereby compromising the membrane’s barrier performance. The ion selectivity of the membranes is graphically presented in Table S2. Both the PVDF-g-IL and PVDF-g-IL/SGO composite membranes exhibit selectivity more than double that of Nafion 115, despite the latter’s significantly higher conductivity. This enhanced performance mainly stems from the exceptional vanadium-blocking capability of the PVDF-g-IL/SGO series. Consequently, these materials demonstrate considerable promise for achieving exceptional performance in vanadium redox flow batteries.

### 3.3. VRFB System Performance

**Figure 6:**
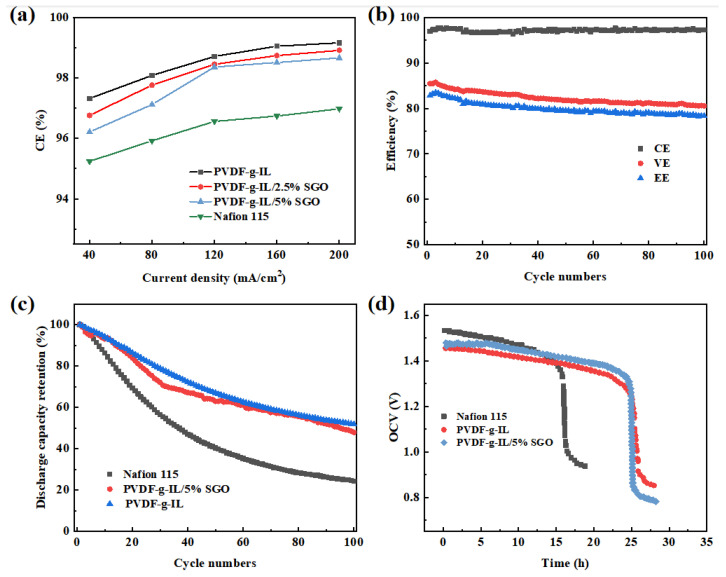
(**a**) The CEs of the PVDF-g-IL, PVDF-g-IL/SGO membranes and Nafion115; (**b**) The efficiencies of the cell equipped with the PVDF-g-IL/5%SGO membrane in the cycling test; (**c**) The discharge capacity retention and (**d**) OCV of the VRFB equipped with the PVDF-g-IL and PVDF-g-IL/5%SGO membranes and Nafion115.

To assess electrochemical characteristics, the performance of our fabricated composite proton-conducting membranes was evaluated and compared against that of the commercial Nafion 115 membrane in VRFB applications, utilizing a current density range of 40 to 200 mA/cm^2^. As depicted in Figure 6a, the CE of the cell was recorded. Every synthesized composite proton-conducting membrane showed superior CE values compared to Nafion 115, suggesting a more effective obstruction of vanadium ion crossover. Among these, the PVDF-g-IL membrane achieved the highest CE, surpassing 98%, which correlates strongly with its very limited vanadium permeability. In contrast, the PVDF-g-IL/SGO hybrid membrane displayed reduced CE relative to the PVDF-g-IL, possibly due to its comparatively lower vanadium ion barrier properties. Additionally, as seen in Figure S8, the EE and VE of the developed composite proton-conducting membranes were marginally inferior to those of Nafion 115.

Cycling stability was examined using a cell equipped with a PVDF-g-IL/5% SGO membrane, running at 100 mA/cm^2^ (Figure 6b). Over 100 cycles, this membrane maintained CE, VE, and EE near 97%, 80%, and 78%, respectively. The slight decrease in energy efficiency and voltage efficiency may be attributed to water electrolysis occurring in the negative electrode compartment (Figure S9). Such consistency also suggests that the IL within the composite did not suffer from substantial dissolution or breakdown and that the SGO filler remained securely embedded. Analysis of the membrane’s mechanical properties before and after cycling tests revealed a marginal reduction in tensile strength (Figure S10). This minimal change underscores the material’s robust mechanical durability. Meanwhile, Figure 6c illustrates the gradual loss in discharge capacity per cycle. By the 100th cycle, both the PVDF-g-IL and PVDF-g-IL/5% SGO membranes retained over 50% of their initial discharge capacity, while the Nafion 115 membrane had declined to 24%. This attenuated capacity fade is mainly ascribed to the membrane’s superior ability to restrict electrolyte diffusion. Consequently, the PVDF-g-IL/5%SGO composite membrane’s markedly lower vanadium permeability, as indicated by its high CE and gradual OCV decay, enables prolonged operational lifetime and diminished electrolyte maintenance needs in long-duration energy storage systems.

The OCV performance of VRFBs assembled using PVDF-g-IL and PVDF-g-IL/SGO composite proton-conducting membranes is depicted in Figure 6d. Experimental data reveal that the initial discharge voltage for the cell incorporating Nafion 115 (1.53 V) exceeds that of cells using the PVDF-based composite proton-conducting membranes. The initial voltage for the Nafion 115 cell is higher, which is attributed to its superior proton conductivity, enabling a faster equilibration to a stable OCV after charging. However, the decisive performance indicator is the subsequent decay rate, which is governed by vanadium ion crossover. The OCV of the VRFB with the Nafion 115 membrane undergoes a rapid decline after sustaining operation for about 15 h. Conversely, batteries constructed with the PVDF-g-IL and PVDF-g-IL/5%SGO composite proton-conducting membranes demonstrate markedly better durability, maintaining an OCV greater than 1.2 V for nearly 25 h, a period substantially longer than that achieved with the Nafion 115 membrane. This sustained voltage performance suggests that VRFBs utilizing Nafion 115 suffer from more pronounced self-discharge phenomena compared to those employing the composite proton-conducting membranes. These OCV findings are consistent with previously obtained permeability data, which indicated that the vanadium ion crossover rate is considerably lower across the composite proton-conducting membranes than through the Nafion 115 membrane. Thus, within VRFB applications, the PVDF-g-IL and PVDF-g-IL/SGO composite proton-conducting membranes proved to be more effective at suppressing self-discharge.

### 3.4. Cost and Prospect Assessment

To clarify the cost of the membranes in this work, a simple calculation of the cost of the grafted membranes was carried out. When preparing PVDF-g-IL/2.5%SGO membranes, 50 g of grafting powder and 2.5 g of SGO are required per square meter of membrane area. Thus, the amount of IL required would be 50/1.25/20% × 30% = 60 g, where 20% is the reaction solid–liquid ratio and 30% is the monomer mass concentration. Correspondingly, the cost of critical materials is listed in Table S3. Besides, the estimation of the cost of the radiation technique was conducted as well. At present, the radiation technique has been widely used in the industry as it is economical for large-scale production. In China, the cost of industrial radiation operation is about $500–700 per ton. Therefore, the cost of preparing a membrane with an area of 1 m^2^ in terms of radiation should be $500–700 per ton × 52.5 g = $0.03–0.04. Overall, the cost of the PVDF-g-IL/2.5%SGO membrane with an area of 1 m^2^ is about $118.73–118.74, which is much lower than that of Nafion115 ($600 m^−2^).

The promising functional properties demonstrated here warrant a discussion on translational challenges. The radiation grafting step, central to our synthesis, presents a scalability and cost consideration compared to simpler casting processes; however, industrial-scale electron accelerators exist for polymer modification. Large-area, uniform membrane fabrication would require transitioning from laboratory casting to continuous processes. A definitive lifecycle cost comparison with Nafion remains a complex task requiring long-term durability data beyond this study. Nevertheless, the high ion selectivity of our composites suggests a potential pathway to reduced electrolyte maintenance and longer operational lifespan, which could offset initial manufacturing costs. Future work must therefore focus on (i) optimizing the grafting process for efficiency, (ii) validating long-term chemical and mechanical stability under real cycling conditions, and (iii) prototyping larger membrane areas to assess manufacturing uniformity.

## 4. Conclusions

The core objective of this work was to engineer a hybrid anion-exchange membrane that deliberately balances the classic trade-off between ionic conductivity and vanadium selectivity in VRFBs. This was achieved through a rational architectural design: PVDF matrices exhibit fundamental, outstanding vanadium barrier properties through irradiation-induced grafting of imidazolium ionic liquids, while strategically incorporated SGO nanosheets introduce controlled proton transport pathways to mitigate the high area-specific resistance typical of conventional AEMs in acidic media. The success of this design was verified through FTIR and XPS characterization of the membrane. Against the benchmark Nafion 115, the synthesized PVDF-g-IL/SGO composite proton-conducting membranes demonstrate comparable ion exchange capacity, superior mechanical strength, and a markedly lower vanadium ion permeability. Consequently, the VRFB single cell employing this membrane achieved high CE and exceptional capacity retention over cycling, directly attributable to the effective suppression of vanadium crossover. Critically, the introduction of SGO successfully enhanced proton conductivity to a level where the resulting VE and EE are only marginally lower than those of Nafion 115. This demonstrates that our membrane architecture successfully decouples the ion transport mechanisms, maintaining high selectivity via the framework while enabling sufficient proton conduction to keep ohmic losses low. This synergistic effect, combined with a potentially cost-favorable position, positions this composite membrane as a viable and promising high-performance alternative for durable VRFB applications.

## Figures and Tables

**Table 1:** Oxidation resistance of PVDF-g-IL/SGO and Nafion 115 membranes after immersed by 1.5 M VO_2_^+^ solutions for 7 days.

Membrane	Weight Loss (%)	Reduction of VO_2_^+^ to VO^2+^ (%)
PVDF-g-IL	3.8	3.2
PVDF-g-IL/0.5%SGO	4.2	3.6
PVDF-g-IL/1%SGO	4.1	3.5
PVDF-g-IL/2.5%SGO	3.8	3.2
PVDF-g-IL/5%SGO	4.0	3.4
Nafion 115	0.56	1.3

**Table 2:** Water uptake rate, acid uptake rate and volume swelling rate.

	Thickness	Water Uptake	Acid Uptake	Swelling Rate
PVDF-g-IL	118 μm	7.54 ± 0.07%	4.15 ± 0.04%	11.24 ± 0.11%
PVDF-g-IL/0.5%SGO	123 μm	7.70 ± 0.13%	5.56 ± 0.05%	11.29 ± 0.09%
PVDF-g-IL/1%SGO	121 μm	8.21 ± 0.11%	5.84 ± 0.07%	11.42 ± 0.13%
PVDF-g-IL/2.5%SGO	119 μm	9.75 ± 0.08%	6.52 ± 0.10%	12.51 ± 0.11%
PVDF-g-IL/5%SGO	118 μm	10.34 ± 0.10%	8.04 ± 0.07%	12.85 ± 0.14%
Nafion 115	120 μm	25.96 ± 0.26%	33.21 ± 0.18%	10.06 ± 0.09%

Data are presented as mean ± standard deviation (*n* = 3).

**Table 3:** Permeability of vanadium ions through the PVDF-g-IL and PVDF-g-IL/SGO membranes and Nafion115.

Membrane	P (×10^−7^ cm^2^/min)
Nafion 115	11.5
PVDF-g-IL	0.95
PVDF-g-IL/0.5%SGO	1.27
PVDF-g-IL/1%SGO	1.38
PVDF-g-IL/2.5%SGO	1.59
PVDF-g-IL/5%SGO	2.02

## Data Availability

The data presented in this study are available on request from the corresponding author.

## Supplementary Materials

The following supporting information can be downloaded at: https://www.mdpi.com/article/10.3390/membranes16020055/s1, Figure S1: The test mold of proton conductivity; Figure S2: The FTIR spectra of the neat PVDF powder and membrane, and the PVDF-g-IL powder and membrane; Figure S3: The ^1^H NMR spectrum of the PVDF-g-IL powder; Figure S4: Cross-sectional SEM image of PVDF-g-IL/5% SGO membrane; Figure S5: Tensile strength and elongation at break of PVDF-g-IL and PVDF-g-IL/SGO membranes; Figure S6: FTIR (a) and ionic conductivity (b) of PVDF-g-IL series membranes before and after immersion in Fenton’s reagent; Figure S7: (a) The EIS curves and (b) the enlarged EIS curves and the equivalent circuit; Figure S8: The EEs (a) and VEs (b) of the PVDF-g-IL, PVDF-g-IL/SGO membrane and Nafion115; Figure S9: The volume of negative electrolytes before and after the cycle test of the PVDF-g-IL/5%SGO membrane; Figure S10: Mechanical properties of PVDF-g-IL/5%SGO membranes before and after cycling; Table S1: Comparison of nanomaterials-modified membranes for VRFB single cell [35,40,41,42,43]; Table S2: Selectivity of PVDF-g-IL and PVDF-g-IL/SGO membranes and Nafion115; Table S3: The cost of the material and radiation for preparing 1 m^2^ membrane.
