# Supplementary material for: Sulfonated Graphene Oxide Doped Imidazolium-Functionalized PVDF Ion Exchange Membrane with Enhanced Ion Conductivity
Source: Membranes (Basel). 2026 Jan 31;16(2):55. doi: 10.3390/membranes16020055 (PMC12943753; doi:10.3390/membranes16020055)
Supplement: Supplementary file 1 [file membranes-16-00055-s001.zip › membranes-4102320-supplementary.pdf]

Supporting Information

Sulfonated Graphene Oxide Doped Imidazolium-Functionalized PVDF Ion Exchange Membrane with Enhanced Ion Conductivity

Jiangtao Yu [1,2], Wenkang Li [3], Wei Niu [1], Manman Zhang [3], Junqing Bai [1], Pengtao Li [1], Liang Wang [1], Yuqing Cui [1], Shuanfang Cui [1], Xueyan Que [1,*], Jun Ma [2,*] and Long Zhao [3,*]

1 Yangling Hesheng Irradiation Technologies Co., Ltd., Xianyang 712000, China; yujiangtao@ssnhsit.com (J.Y.); niuwei@ssnhsit.com (W.N.); baijunqing@ssnhsit.com (J.B.); lipengtao@ssnhsit.com (P.L.); wangliang@ssnhsit.com (L.W.); cuiyuqing@ssnhsit.com (Y.C.); cuishuanfang@ssnhsit.com (S.C.)

2 School of Nuclear Science and Technology, University of Science and Technology of China, Hefei 230026, China

3 State Key Laboratory of Advanced Electromagnetic Technology, School of Electrical and Electronic Engineering, Huazhong University of Science and Technology, Wuhan 430074, China; liwenkang@hust.edu.cn (W.L.); zhangmm@hust.edu.cn (M.Z.)

* Correspondence: quexueyan@ssnhsit.com (X.Q.); majun0502@ustc.edu.cn (J.M.); zhaolong@hust.edu.cn (L.Z.)

Section S1. Method of tensile test

To begin, the membrane must be completely dried. This is achieved by placing it in a drying oven at 80 °C for a period of 24 hours, until the mass discrepancy between two successive weighings falls below 0.2 mg. To enhance measurement accuracy, the membrane's thickness was gauged at three distinct locations. Specimens were then precision-cut into a dumbbell shape, with care taken to avoid edge defects during this process. To preclude slippage in the testing apparatus, double-sided adhesive was applied to both ends of each sample. For the tensile tests performed in this study, a 100 N capacity load cell was used for all measurements. The test was performed at a constant speed of 50 mm min^{-1}. For every film variety, five separate specimens were tested to ensure reliability, with validation contingent upon the fracture occurring within the central gauge section.

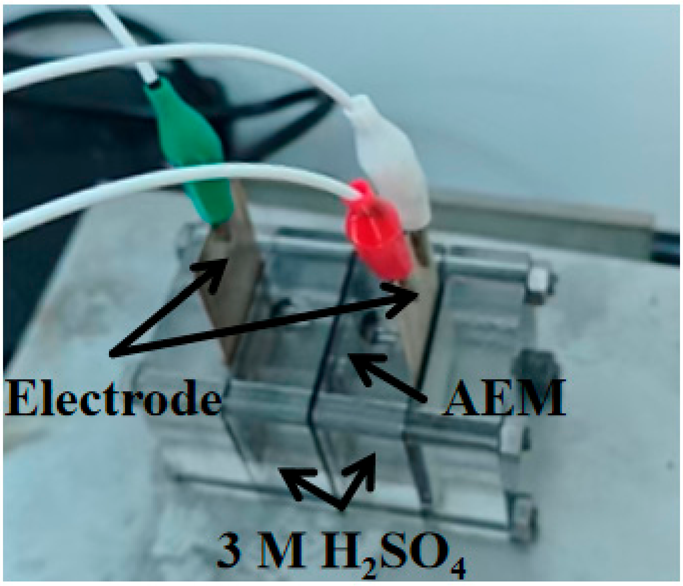

Figure S1. The test mold of proton conductivity.

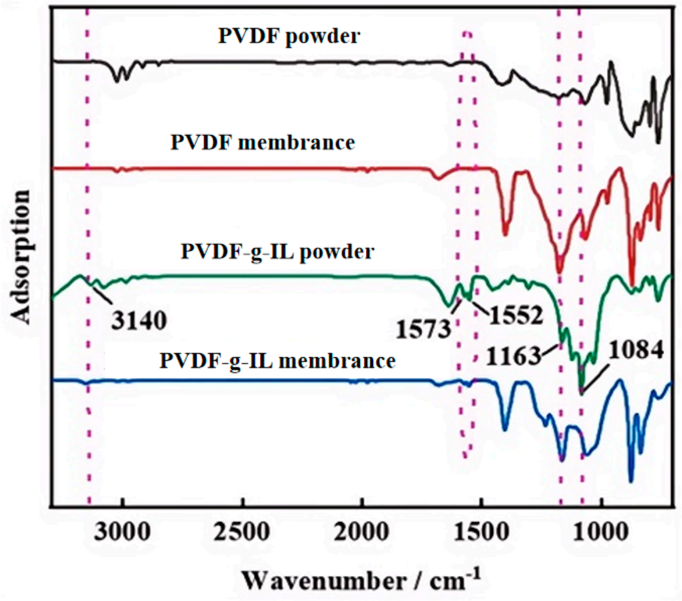

Figure S2. The FTIR spectra of the neat PVDF powder and membrane, and the PVDF-g-IL powder and membrane.

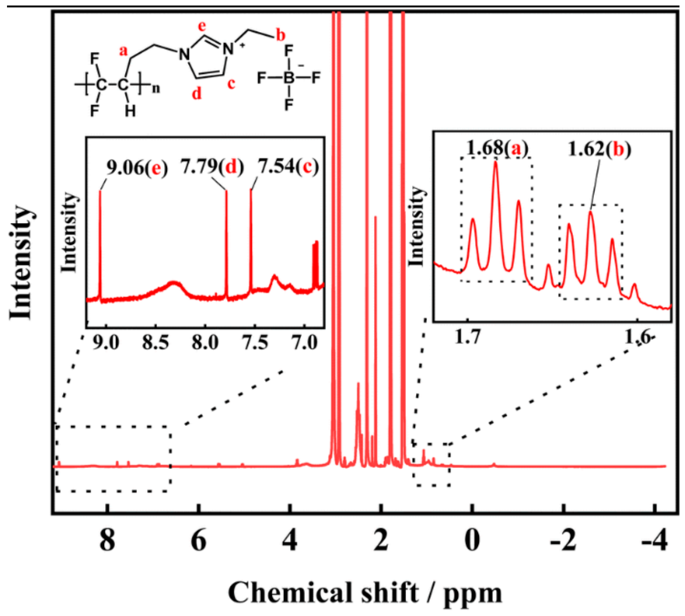

Figure S3. The ^{1}H NMR spectrum of the PVDF-g-IL powder.

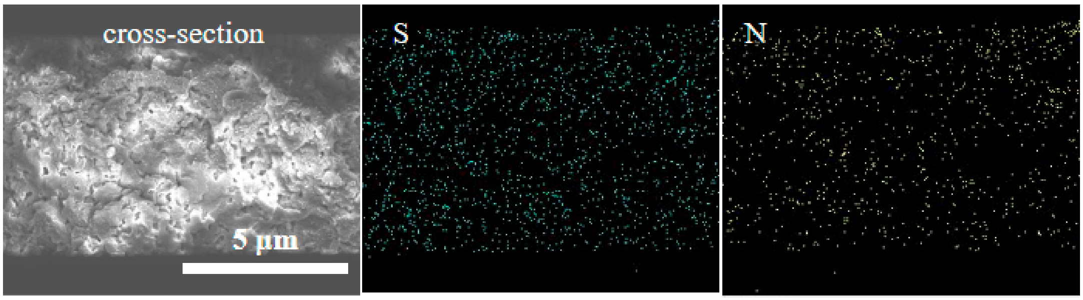

Figure S4 Cross-sectional SEM image of PVDF-g-IL/5% SGO membrane

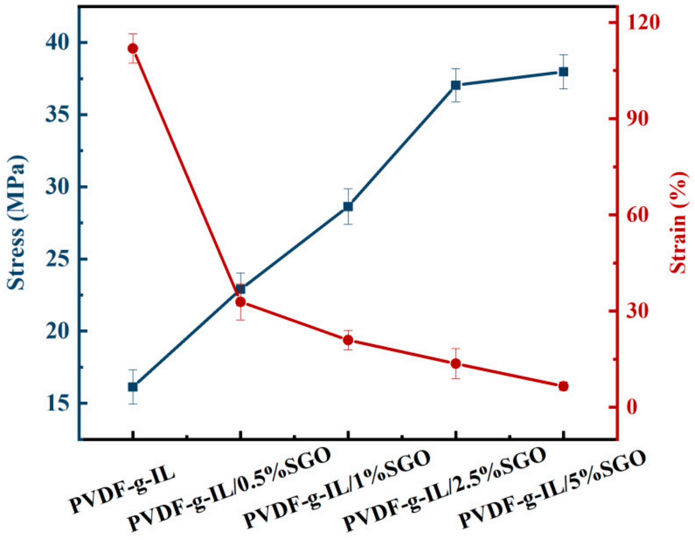

Figure S5. Tensile strength and elongation at break of PVDF-g-IL and PVDF-g-IL/SGO membranes.

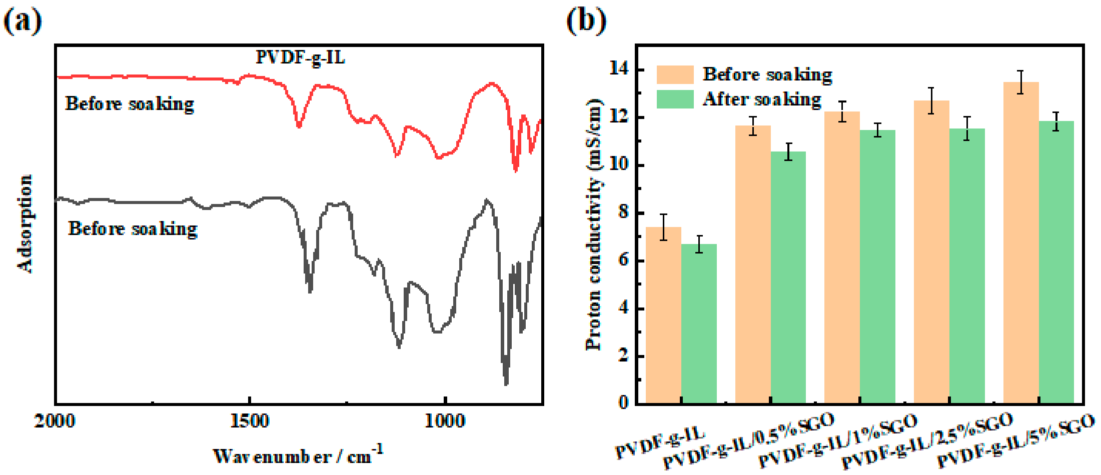

Figure S6. FTIR (a) and ionic conductivity (b) of PVDF-g-IL series membranes before and after immersion in Fenton's reagent.

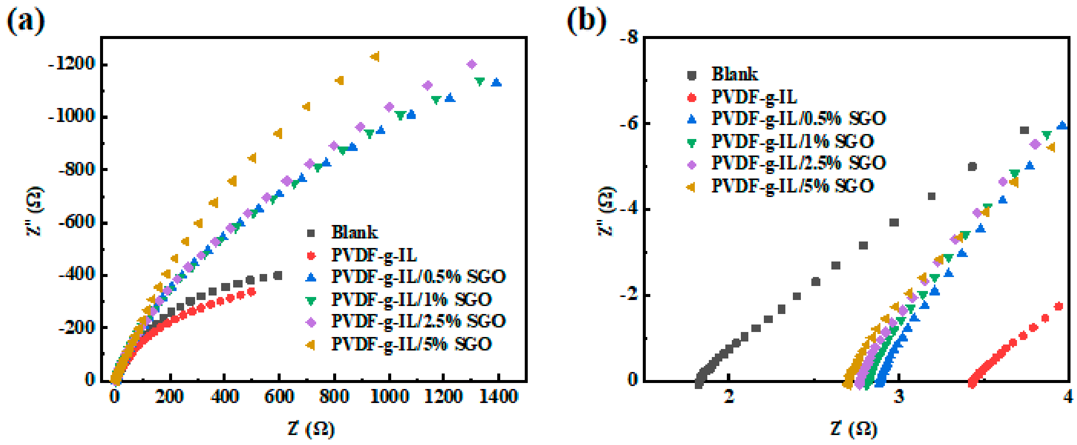

Figure S7. (a) The EIS curves and (b) the enlarged EIS curves and the equivalent circuit.

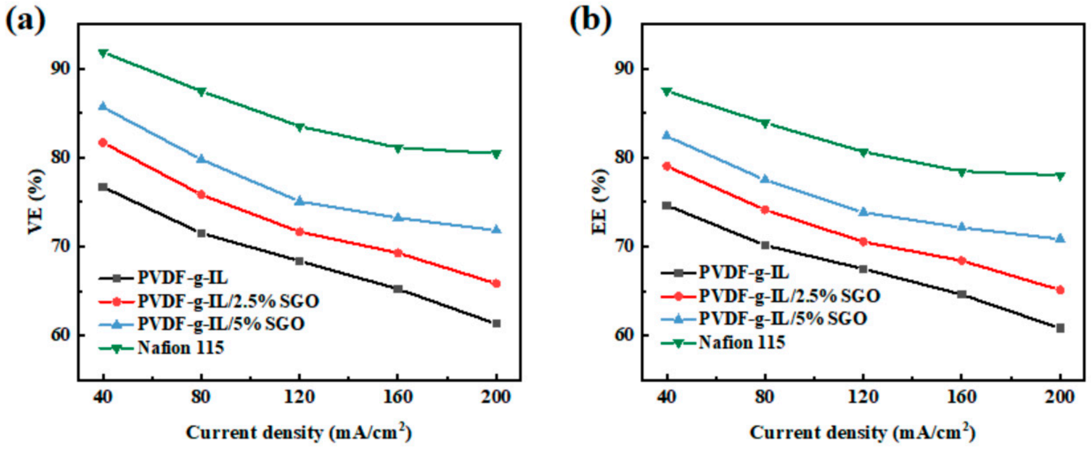

Figure S8. The EEs (a) and VEs (b) of the PVDF-g-IL, PVDF-g-IL/SGO membrane and Nafion115.

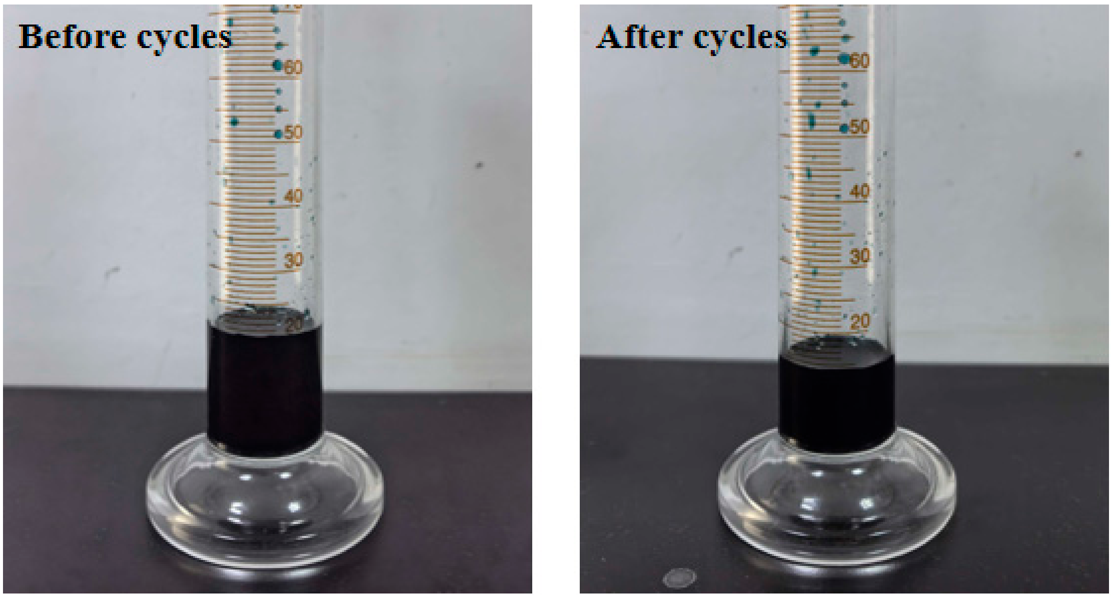

Figure S9. The volume of negative electrolytes before and after the cycle test of the PVDF-g-IL/5%SGO membrane.

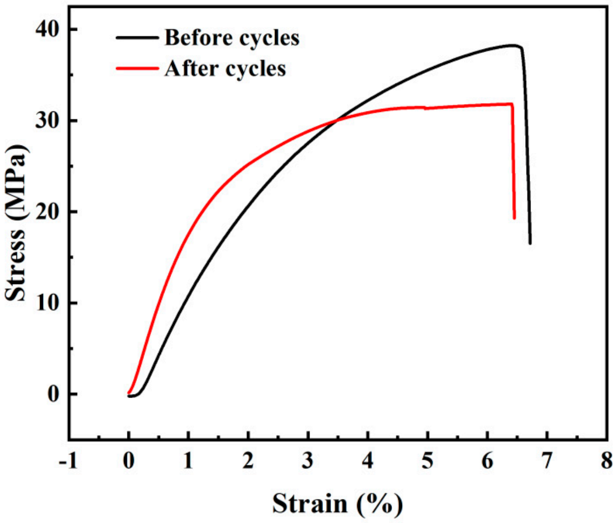

Figure S10. Mechanical properties of PVDF-g-IL/5%SGO membranes before and after cycling

Table S1. Comparison of nanomaterials-modified membranes for VRFB single cell.

Membrane	VO^{2+} permeability (P) (10^{-7} cm^2/min)	Conductivity (σ) (mS/cm)	CE (%)	EE (%)	Ref.
N-117/SiO_2-SO_3H	1.3	34.82	94	82	[1]
Nafion/TiO_2	67.2	-	88.8	71.5	[2]
rNafion/GO	8.1	29.3	96	85	[3]
5%@Nafion/SiO_2	2.5	130	93.5	83.9	[4]
PVDF-g-IL	0.95	9.05	97.6	73	[5]
PVDF-g-IL/5%SGO	2.02	15.47	96.2	82.4	This work

Table S2. Selectivity of PVDF-g-IL and PVDF-g-IL/SGO membranes and Nafion115.

Membrane	S (×10^7 (mS min)/cm^3)
Nafion 115	3.79
PVDF-g-IL	7.41
PVDF-g-IL/0.5%SGO	8.15
PVDF-g-IL/1%SGO	8.38
PVDF-g-IL/2.5%SGO	7.96
PVDF-g-IL/5%SGO	7.87

Table S3. The cost of the material and radiation for preparing 1 m^2 membrane.

Material	Unit price	Required amount	Cost
[C_2VIm][BF_4]	$72.8 / 100g	60g	$43.68
PVDF	$37.8 / kg	40g	$1.52
SGO	$27.8 / 1g	2.5g	$69.5
Ethanol	$1 / 500ml	1000ml	$2
NMP	$4 / L	500ml	$2
Radiation	$500-700 / ton	52.5 g	$0.03-0.04
	Overall		$118.73-118.74

Reference

1. Huang, S.L.; Yu, H.F.; Lin, Y.S. Modification of Nafion® Membrane via a Sol-Gel Route for Vanadium Redox Flow Energy Storage Battery Applications. *J Chem* 2017, *2017*, doi:10.1155/2017/4590952.
2. Wang, N.; Peng, S.; Lu, D.; Liu, S.; Liu, Y.; Huang, K. Nafion/TiO_2 Hybrid Membrane Fabricated via Hydrothermal Method for Vanadium Redox Battery. *Journal of Solid State Electrochemistry* 2012, *16*, 1577–1584, doi:10.1007/s10008-011-1560-z.
3. Yu, L.; Lin, F.; Xu, L.; Xi, J. A Recast Nafion/Graphene Oxide Composite Membrane for Advanced Vanadium Redox Flow Batteries. *RSC Adv* 2016, *6*, 3756–3763, doi:10.1039/c5ra24317c.
4. Zeng, S.; Zeng, L.; Wang, R.; Guo, W.; Tang, H. Effect of Elevated Temperature Annealing on Nafion/SiO_2 Composite Membranes for the All-Vanadium Redox Flow Battery. *Polymers (Basel)* 2018, *10*, doi:10.3390/polym10050473.
5. Wang, Z.; Jiang, J.; Dong, Z.; Song, Y.; Zhao, L. Radiation Synthesis of Imidazolium Ionic Liquid Grafted PVDF as the Anion Exchange Membrane for Vanadium Redox Flow Batteries. *New Journal of Chemistry* 2023, *47*, 8013–8021, doi:10.1039/d2nj05386a.
